# DUSP1 Is a Potential Marker of Chronic Inflammation in Arabs with Cardiovascular Diseases

**DOI:** 10.1155/2018/9529621

**Published:** 2018-12-13

**Authors:** Abdelkrim Khadir, Sina Kavalakatt, Mohammed Dehbi, Monira Alarouj, Abdullah Bennakhi, Ali Tiss, Naser Elkum

**Affiliations:** ^1^Research Division, Dasman Diabetes Institute, Kuwait; ^2^Diabetes Research Centre, QBRI, Hamad Bin Khalifa University, Doha, Qatar; ^3^Sidra Medicine, Doha, Qatar

## Abstract

**Background:**

Cardiovascular disease (CVD) risks persist in patients despite the use of conventional treatments. This might be due to chronic inflammation as reflected in epidemiological studies associating circulating low-grade inflammatory markers with CVD recurrent events. Here, we explored this potential link by assessing plasma dual-specificity phosphatase 1 (DUSP1) levels and comparing them to high-sensitivity CRP (hsCRP) and oxidized low-density lipoprotein (oxLDL) levels and their associations to conventional CVD risk factors in confirmed CVD patients.

**Methods:**

Human adults with reported CVD (*n* = 207) and controls (*n* = 70) living in Kuwait were used in this study. Anthropometric and classical biochemical parameters were determined. Plasma levels of DUSP1, oxLDL, and hsCRP were measured using human enzyme-linked immunosorbent assay kits.

**Results:**

DUSP1 and hsCRP plasma levels and their least square means were higher in CVD cases, while oxLDL plasma levels were lower (*p* < 0.05). Multivariate logistic regression analysis showed that DUSP1 and hsCRP are independently associated with CVD in the studied population, as reflected by 2-fold and 1.5-fold increased risks with increased levels of DUSP1 and hsCRP, respectively. In our study, DUSP1 levels were found to be associated with CVD despite statin treatment and diabetes status (*p* < 0.05), whereas hsCRP mainly correlated with obesity markers.

**Conclusions:**

Circulating DUSP1 might be a predictor of chronic subclinical inflammation and residual risk in CVD patients, whereas our data suggest that the association between hsCRP and CVD is largely accounted for adiposity risk factors.

## 1. Background

Incidences of diabetes and obesity continue to increase and so do their complications. One of these complications, cardiovascular disease (CVD), continues to be a leading cause of mortality worldwide [1]. Despite the establishment of classical risk factors involved and the availability of conventional drug treatments, many CVD patients experience potential events associated with residual CVD risk [2]. Immune system imbalance and metabolic stress and their crosstalk play a pivotal role in the initiation and progression of the disease [3]. Indeed, it has become clear that in a large proportion, atherosclerosis, a major cause of several CVDs, is related to a chronic inflammation process involving a complex network of multiple cells and inflammatory and metabolic stress mediators [4]. Therefore, novel biomarkers for stratification and identification of CVD risk factors, beyond the traditional ones, are needed to improve disease management.

At the molecular level, mitogen-activated protein kinase (MAPK) signaling pathways play a prominent role as modulators of stress and inflammatory responses in CVD, particularly during the atherogenesis process [5]. Inactivation of MAPKs by dual-specificity phosphatase 1 (DUSP1), also called mitogen-activated protein kinase phosphatase (MKP-1), was recently shown to be increased in circulating blood cells of CVD patients even after treatment with conventional drugs, such as statins [6]. However, the question as to whether DUSP1 influences atherogenesis and thereby CVDs is still controversial and remains to be clarified [7]; indeed, DUSP1 has been reported to be both antiatherogenic [8] and proatherogenic [9]. Likewise, DUSP1 has been linked to the atherosclerosis process in various animal and cell models of CVD [7]. Furthermore, we have shown circulating DUSP1 to be elevated in the plasma of obese subjects and inversely correlated with circulating HDL, an independent risk factor for CVDs [10].

On the other hand, oxidized low-density lipoprotein (oxLDL) is emerging as a potential biomarker for CVD risk, and its levels are higher in CVD subjects [11]. Circulating oxLDL levels have been reported to be associated with obesity, diabetes, and metabolic syndrome [12, 13], all of which increase the CVD risk. Several studies have focused on strategies for reducing circulating oxLDL levels using different statins, which exhibited variation in their abilities to reduce circulating oxLDL levels [14]. Nevertheless, the usefulness of oxLDL as a clinical biomarker is yet to be validated. C-reactive protein (CRP) is an acute-phase and nonspecific marker of inflammation, produced in liver in response to several cytokines [15]. Interleukin 6 is the most potent driver of CRP production in response to infection, trauma, and atherosclerosis. In the latter disease, CRP directly binds to oxLDL and is present within lipid plaques [16]. Currently, high-sensitivity CRP (hsCRP) is used as biomarker for CVD risk prediction [17]. For instance, and based on evidence from the JUPITER trial, hsCRP measurement has been integrated into the Reynolds risk scoring system to predict CVD risk. However, several recent reports have generated some controversy around data regarding its pertinence as a reliable biomarker in atherosclerosis [18]. Indeed, despite several population-based studies in subjects free of known CVD have shown the superiority of hsCRP above standard risk factors for risk prediction [19], other studies reported very limited contribution of hsCRP to CVD risk prediction [20]. In addition, Animal and human genetic reports have questioned the direct atherogenic effects of hsCRP, highlighting the lack of causal relationship between hsCRP and atherosclerosis [18, 21]. Furthermore, only weak association was observed for hsCRP with the carotid intima-media thickness in the absence of obesity [22].

Morbidity and mortality from CVDs differ significantly, in part based on ethnicity, and this is likely related to differences in risk factors [23]. There may be ethnic differences in the levels of inflammation, which may be partially related to demographic, lifestyle, or genetic factors that need further investigations. We previously showed that despite their apparent healthier status, Arab females are at higher risk of CVD complications due to their increased oxidative stress [24]. In addition, the population of the Gulf region, including Kuwait, is experiencing a high prevalence of metabolic syndrome, obesity, and diabetes due to rapid socioeconomic changes that have increased the panoply of CVD risks [25]. Hence, it is of clinical importance to assess the status of these markers as signatures of CVD in the Arab population living in Kuwait, particularly when chronic inflammation remains unresponsive to standard drugs in many subjects. From this perspective, the present study was undertaken to (i) assess the levels of circulating DUSP1, oxLDL, and hsCRP in a CVD-confirmed population from Kuwait as compared with matched controls and to (ii) evaluate the association of these levels with established CVD risk factors.

## 2. Materials and Methods

### 2.1. Study Participants

In the current study, subjects with reported CVD events were extracted from the Kuwait Diabetes Epidemiology Program (KDEP) that was conducted between June 2011 and August 2012 at the Dasman Diabetes Institute (DDI) to estimate the prevalence of noncommunicable diseases among the resident population of Kuwait [26]. The current study included 207 Arab adults with reported CVD event (cases) and 70 apparently healthy with no reported chronic condition (controls). The study was approved by the Ethical Review Committee at DDI. Data extracted for anthropometric and physical measurements mainly included body weight, height, waist circumference, and blood pressure (BP). Blood sample measurements included fasting blood glucose (FBG) as well as lipid profile, triglycerides (TG), total cholesterol (TC), low-density lipoprotein (LDL), and high-density lipoprotein (HDL).

### 2.2. Circulating Inflammatory Marker Measurements

Circulating plasma levels of DUSP1 and oxLDL were determined using the human DUSP1 and oxLDL enzyme-linked immunosorbent assay (ELISA) kits (EIAab, Wuhan, China), and hsCRP levels were measured using a high-sensitivity CRP “hsCRP” ELISA kit (BioVendor, Asheville, NC). Assays were performed as per the manufacturers' instructions.

### 2.3. Statistical Analysis

Descriptive statistics are presented as means ± standard deviation (SD) for continuous variables or as numbers and percentages for nominal/categorical variables. Student's *t*-test and chi-square test were used to evaluate differences between continuous and categorical variables, respectively. Spearman's correlation coefficients were estimated to determine associations between levels of DUSP1, hsCRP, and oxLDL and various anthropometric and metabolic parameters. Logistic regression analysis was performed to estimate odds ratios (ORs) and to examine the predictive effect of each factor on CVD risk. ORs and their 95% confidence intervals (95% CIs) for associated factors were estimated. Research Electronic Data Capture (REDCap) was used for data collection and management. All statistical assessments were two sided and considered significant at *p* < 0.05. All analyses were performed using SAS (version 9.4; SAS Institute, Cary, NC).

## 3. Results

### 3.1. Characteristics and Biochemical Markers of Study Population

The anthropometric features and medical status of the study population are displayed in Table 1. Cases were more aged than controls (*p* < 0.0001), and more than half of them were diabetic (67.3%). Furthermore, most CVD cases had experienced a vascular complication that resulted in coronary or peripheral artery disease (65%) as compared with 22% diagnosed with heart failure and 10% with a stroke event. Furthermore, more than 50% of cases were treated with statins and blood pressure-lowering medications, and approximately 20% were taking antiaggregant and *β*-blockers. In addition, only 3% of patients had undergone coronary artery bypass graft. We observed significantly higher levels of the traditional circulating metabolic markers FBG, HbA1c TG, and systolic blood pressure (SBP; *p* < 0.01) but not of LDL and TC among cases compared with controls. The observed unexpected low levels of TC and LDL among cases may be attributable to statin treatment. Control subjects had higher levels of good cholesterol HDL. With respect to nonclassical markers, DUSP1 plasma levels were significantly higher in the cases, as reflected by both the median and range of DUSP1 (*p* = 0.02), whereas oxLDL levels were significantly lower in the patient group (*p* = 0.0003; Table 1). Finally, hsCRP displayed a significantly higher median in the CVD group (*p* = 0.04).

### 3.2. Correlation of DUSP1, oxLDL, and hsCRP with Clinical and Metabolic Parameters

We applied Spearman's rank test to determine correlations between circulating levels of DUSP1, oxLDL, and hsCRP proteins and the physical, clinical, and biochemical parameters of the participating subjects. In the control group, there was a statistically significant positive correlation between DUSP1 levels and all obesity indicators and SBP (Table 2). However, in the CVD patient group, significantly negative correlations were observed with age, waist circumference, HDL, and oxLDL. On the other hand, and as expected, hsCRP displayed a positive correlation with the anthropometric parameter of adiposity in both groups. Finally, oxLDL exhibited more correlations in the control group, in particular with BP wherein a strong negative correlation was observed. Lipid profiles unexpectedly showed positive and negative correlations with HDL and TG, respectively (Table 2).

### 3.3. Factors Associated with CVD Risk

**Figure 1:**
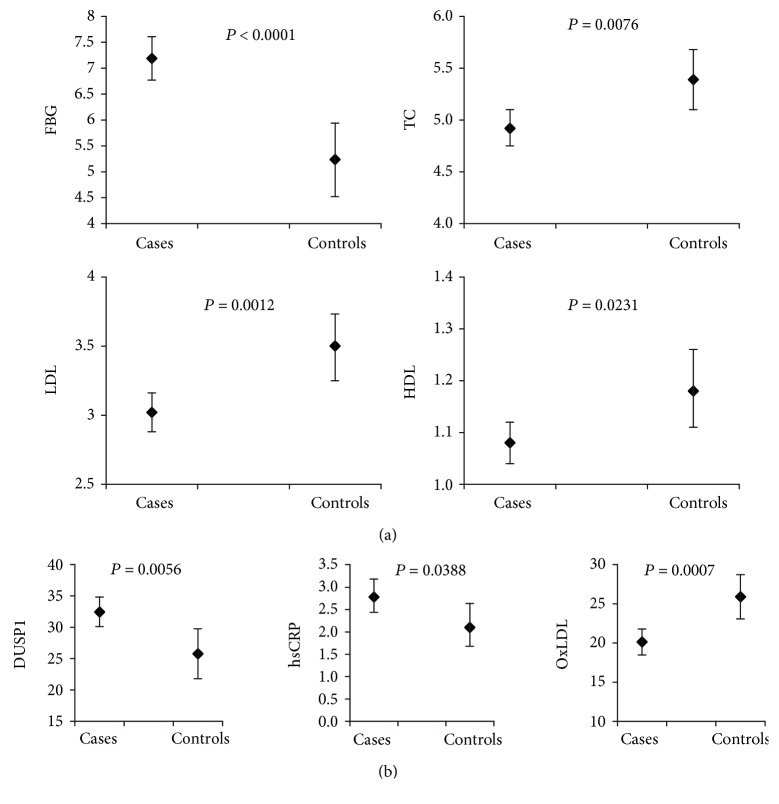
Least square means analysis of various CVD (a) conventional and (b) nonconventional markers.

We next investigated the strength of the abovementioned markers with respect to discriminating CVD patients from the control group using least square means analysis. As expected, the classical CVD risk markers FBG, LDL, TC, and HDL were able to significantly discriminate between CVD cases and controls in the study population (Figure 1(a)). FBG exhibited higher levels in the patients while HDL, LDL, and TC were lower in this group compared with controls. Interestingly and as shown in Figure 1(b), the least square means of DUSP1 and hsCRP were significantly higher in CVD subjects and oxLDL was lower, which agrees with data displayed in Table 1.

We also performed multivariate logistic regression analysis for independent CVD risk predictors. In an initial analysis, FBG was clearly and independently associated with CVD, as reflected by a 5-fold increase in CVD risk with increased levels of glucose (data not shown); this agreed with the data reported in Table 1 and Figure 1. When FBG was excluded from the analysis, DUSP1 was the most significant independent factor for predicting CVD risk (OR = 2.277, *p* = 0.0328; Table 3). Similarly, hsCRP displayed a significantly higher OR for CVD (OR = 1.511, *p* = 0.0227). LDL and oxLDL, however, displayed lower ORs (OR = 0.546, *p* = 0.0006 and OR = 0.461, *p* = 0.0104, respectively).

### 3.4. Trend Analysis of CVD Risk Markers Based on Diabetes and Statin Treatments

Statin treatment is known to affect lipid profiles because these molecules are potent inhibitors of cholesterol biosynthesis. Concordantly, our results exhibited abnormal lipid profiles when comparing CVD cases with their matched controls, particularly for TC and LDL, which were unexpectedly lower in the case group. To try to explain this observation, we segregated our study population based on statin treatment and performed analysis of variance (Table 4). This showed that despite the significantly higher average age of the case group treated with statins, the group exhibited significantly lower values of lipid profiles (TC and LDL). More importantly, DUSP1, hsCRP, and oxLDL were also clearly decreased in comparison with the levels in the untreated group. The trends for the other anthropometric and metabolic markers agreed with the results reported in Table 1, including the entire study population.

As expected, when segregating our subjects based on diabetes status (Table 5), the diabetic subjects were significantly older and had higher glycemic indices, SBP, and TGs than nondiabetic subjects with CVD or nondiabetic non-CVD controls. Although hsCRP displayed an increase in patients with CVD and diabetes, oxLDL decreased in these circumstances. DUPS1 levels were still higher in nondiabetic CVD subjects compared with both controls and diabetic CVD subjects.

## 4. Discussion

Besides the traditional markers and clinical indicators for CVD such as age, sex, smoking, lipid profile, and glycemic index, the only established inflammatory biomarker clinically used is hsCRP, although its specificity is being questioned [27–30]. Thus, novel biomarkers with higher specificity and sensitivity are still needed for assessing residual chronic inflammation in CVD patients and for predicting potential future CVD events. The objective of this case-control study was to compare circulating DUSP1 with two nonclassical biomarkers of chronic inflammation in CVD, oxLDL, and hsCRP. More specifically, we compared their abilities to differentiate between individuals with and without reported CVD events to provide information about residual chronic inflammation risk. Our main findings were that (i) circulating levels of DUSP1 and hsCRP were elevated in CVD cases while oxLDL was higher in controls; (ii) as expected, hsCRP correlated with obesity markers, suggesting that the association between hsCRP and CVD is largely accounted for by adiposity risk factors; and (iii) DUSP1 levels are independently associated with CVD despite statin treatment and diabetes status.

Circulating oxLDL has been reported to be a predictor of acute coronary diseases in the general population [31], and oxLDL levels were higher in subjects with established coronary heart disease when compared with controls [32]. Indeed, the oxidative conversion of LDL to oxLDL is a key factor in the pathophysiological process that initiates the progression of atherosclerotic vascular diseases [33, 34]. In line with this, it has been shown that oxLDL is associated with the subclinical atherosclerosis phase [35, 36] and directly promotes plaque rupture [37, 38]. However, we found lower oxLDL levels in the CVD group when compared with the control group and a similar pattern for LDL and TC. Moreover, oxLDL was better correlated with lipid profile in the control group. This might be explained by the heterogeneity of the CVD group due to various treatment regimens because most CVD subjects were being treated with statins and angiotensin-converting enzyme (ACE) inhibitors, which are known to decrease LDL, oxLDL, and BP levels [39–41]. Indeed, when our CVD subjects were segregated, significantly lower levels of oxLDL, LDL, and TC were measured in statin-treated subjects. Furthermore, the latter group had a significantly higher age and more incidences of diabetes compared with the nonstatin or the control group (Tables 4 and 5). Consistent with this, distorted lipid and oxLDL profiles have been reported in previous studies and were suggested to be linked to confounding factors such as diet as well as the size and composition of cholesterol particles, which may affect the oxidation process [42]. Overall, our findings that oxLDL was better correlated with and found at higher levels in the control group and CVD subjects not treated with statin further highlight the potential of using oxLDL as a predictor of CVD events in apparently healthy obese individuals or in CVD risk subjects but not in CVD-treated ones; this is in line with previous studies [43, 44]. Because most non-CVD control subjects were obese, they might also be considered at risk for CVD events and potentially experiencing chronic subclinical inflammation.

On the other hand and among potential cardiovascular inflammatory markers, CRP is an acute-phase product produced predominantly in hepatocytes and emerged as a valuable biomarker in refining CVD risk assessment [17]. Several reports have shown that despite being consistently associated with CVD risk, CRP measurement produces particularly inconsistent results for risk prediction in apparently healthy subjects [18]. However, CRP is also widely used as a nonspecific indicator of inflammation in different pathologic situations. There was a significant increase in hsCRP levels between cases and controls in this study, and we found a strong correlation in both groups between anthropometric parameters of obesity and hsCRP, as previously reported [45]. Furthermore, hsCRP displayed a significant OR and association with CVD even after correcting for various confounding factors such as age, sex, and obesity (Table 3) and after segregating our study population based on statin treatment and diabetes. These results support the potential use of CRP for predicting CVD in apparently healthy individuals as well as in CVD patients. On a note of caution, we did not exclude from our study participants with extreme CRP levels as high CRP levels were predictive of cardiovascular risk [46]. Furthermore, our study mainly used obese subjects and thus the association between elevated CRP levels and CVD might be, at least in part, due to obesity. Thus, the observed CRP levels might have been due to the known low-grade chronic inflammation associated with obesity rather than CVD. Furthermore, the effect of statins on CRP levels was clear, and this might affect the predictive value of CRP in CVD. Nevertheless, our results and those of others [47] reporting the fact that statin therapy decreases hsCRP levels underscore a potential role of inflammation in CVD as a target for intervention in elderly and diabetic individuals.

In our previous work, we have shown that circulating and cellular DUSP1 levels are elevated in nondiabetic obese individuals as compared with normal weight individuals [10]. Here, we report increased circulating levels of DUSP1 in CVD cases in comparison with apparently healthy controls despite both groups being obese. Furthermore, and after adjustment for the usual risk factors using multivariate analyses, DUSP1 exhibited a relatively strong association with CVD (OR = 2.277) despite the possible confounding factors in our study (diabetes and statin treatment). More interestingly, when subjects were clustered by statin treatment or diabetes, the highest circulating levels of DUSP1 were observed in the group without statin treatment and in CVD patients without diabetes. This suggests a better predictive power of DUSP1 in these nontreated groups and potentially those undergoing low-grade inflammation. Indeed, DUSP1, as a negative modulator of MAPKs, has been shown to be a key player in several physiological and pathophysiologic processes, such as the immune response, metabolic diseases, and different types of cancers [48]. Thus, it is not surprising that DUSP1 is associated with the development and progression of CVDs. Moreover, it has been reported that expression levels of DUSP1 in circulating blood cells exhibited a more consistent difference between patients and controls in identifying coronary artery disease history when compared with hsCRP [49].

On the other hand, our observed decrease in circulating DUSP1 levels due to statin treatment disagrees with previous studies. Indeed, Linden and colleagues have reported that these drugs fail to decrease DUSP1 in blood cells of CVD-treated patients (6). This discrepancy might be explained by a difference between circulating and cellular DUSP1 levels, as we have previously observed [10]. Furthermore, the decreased levels of DUSP1 in the CVD diabetic group might be linked with hyperglycemia in these subjects. In line with this, a decreased expression of DUSP1 in the myocardium of streptozotocin-induced diabetic rats has been reported and suggests a role of DUSP1 and MAPKs in the pathophysiology of diabetic cardiomyopathy [50]. Moreover, chronic high glucose-induced insulin resistance is accompanied with marked reductions in DUSP1 induction [51]. Meanwhile, MAPK activation in diabetic glomerular nephropathy has also been shown to be due to suppression of DUSP1 by high glucose [52]. Finally, hyperglycemia is an important epigenetic factor that induces several modifications leading to differential expression of genes involved in diabetes-induced chronic pathologies, such as vascular complications [53], and DUSP1 single-nucleotide polymorphisms and expression levels have been reported to be affected by this process in the morbidly obese and those with metabolic syndrome [54]. In agreement with these observations, our multivariate regression analysis clearly revealed a nonneutral interaction between DUSP1 levels and glucose, thus affecting its association with CVD risk. Indeed, FBG outscored all markers in terms of OR, producing a 5-fold increased risk for CVDs, but DUSP1 was not associated to this risk when FBG was considered in the model. However, when FBG was excluded, DUSP1 independently associated with a more than 2-fold increased risk of CVD.

Interestingly, no correlation between oxLDL and hsCRP was observed in the present study suggesting that the role of these markers in the pathophysiology of atherosclerosis process is not completely linked. However, we observed and we reported here for the first time a negative correlation between oxLDL and DUSP1. Such a relation was shown very recently in human monocytes treated with oxLDL wherein DUSP1 was repressed, and thus, p38 has been activated [55].

Overall and despite our report here that DUSP1 might be a potential predictor of continuous inflammation processes in CVD patients, the current study has some limitations that deserve consideration. The cross-sectional nature of this study does not allow for the determination of causality, i.e., whether the increase in DUSP1 is a cause or a consequence of CVDs. Other limitations of our study included the patient selection process, which might have introduced some confounding variables, such as environmental factors and the fact that some clinical data on patients were missing, including full medical history. In addition, and despite our adjustment for some confounders, we cannot exclude the possibility that the observed associations in our study can be explained by other residual confounding. Moreover, possible impact of therapy in our study cohort may not be ruled out as confounder. Finally, our results might not be generalizable to other populations because our study included only Arab population living in Kuwait. In perspective, further studies on the role and regulation of DUSP1 are required before drawing definitive conclusions on the predictive value and association between this marker and CVD. The role, if any, of secreted DUSP1 is yet to be elucidated due to the paucity of data on its circulating form compared with DUSP1 in cells and tissues. Moreover, establishing cut-off values and ranges of DUSP1 in plasma from normal and CVD patients with various phenotypes are needed in large prospective cohort studies.

In conclusion, to the best of our knowledge, our study is the first to assess DUSP1 potential in predicting the development of CVD in the general population as compared to oxLDL and hsCRP in an Arab population. Moreover, our study used a high-risk group where epidemiological data from Arabian Gulf countries indicated a high rate of obesity and diabetes [56]. Our data suggest that high circulating DUSP1 levels reflect a chronic subclinical inflammation and underlying residual CVD risks even in the treated population. In addition, oxLDL might be a good marker for the prediction of CVD events in apparently healthy individuals. Prospective and larger studies are needed to clarify the rationale for DUSP1's role as a marker of residual inflammation in CVDs, as was previously done for hsCRP. Finally, CRP is an established nonspecific prognostic inflammatory biomarker for patients with atherosclerosis. Some other inflammatory markers exhibit poor predictive power when used alone. Therefore, a panel of inflammatory biomarkers may be required to predict residual risk and long-term cardiovascular outcome after an event, with more accuracy.

## Figures and Tables

**Table 1:** Study population characteristics.

Parameter	Cases *n* = 207	Controls *n* = 70	*p* value
Anthropometric			
Age (years)	54.5 ± 11.7	47 ± 11.5	**<0.0001**
Gender (M/F)	132/75	36/34	0.0677
Weight (kg)	85.8 ± 17	85.6 ± 18	0.9245
Waist (cm)	103.3 ± 13.8	99.6 ± 12.8	0.0523
Hip (cm)	109.3 ± 12.0	110 ± 11.0	0.4631
BMI (kg/m^2^)	31.4 ± 5.96	30.9 ± 5.36	0.5621
Risk factors			
Smoking	24.5%	28%	0.2750
Diabetes	67.3%	—	
Type of CVD and intervention			
Coronary and peripheral artery disease	65.0%	—	
Heart failure	22.0%	—	
Stroke	10%	—	
Coronary artery bypass graft	3%	—	
Medication			
Statins	54%	—	
Antiplatelets	13%	—	
*β*-Blockers	7%	—	
Blood pressure treatment	50%	—	
Blood pressure			
SBP (mmHg)	136.9 ± 20.1	129 ± 17.7	**0.0046**
DBP (mmHg)	77.8 ± 12.0	79.3 ± 11.8	0.3431
Metabolic markers			
FBG (mmol/l)	7.35 ± 3.48	4.91 ± 0.38	**<0.0001**
HbA1c (%)	7.30 ± 2.12	5.60 ± 0.46	**<0.0001**
TC (mmol/l)	4.87 ± 1.29	5.42 ± 0.97	**0.0002**
TG (mmol/l)	1.76 ± 1.43	1.46 ± 0.85	**0.0350**
HDL (mmol/l)	1.10 ± 0.35	1.21 ± 0.37	**0.0207**
LDL (mmol/l)	2.97 ± 1.06	3.54 ± 0.80	**<0.0001**
Circulating inflammatory markers			
DUSP1 (ng/ml)	27.85 (11.25-139.08)	25.14 (0.33-68.65)	**0.0203**
hsCRP (mg/l)	3.12 (0.09-11.85)	2.37 (0.20-10.50)	**0.0426**
oxLDL (*μ*g/l)	16.0 (0.03-61.21)	18.6 (5.19-60.25)	**0.0003**

Data are presented as mean ± SD. ^∗^*p* value shows the differences between cases and controls. BMI: body mass index; SBP: systolic blood pressure; DBP: diastolic blood pressure; FBG: fasting blood glucose; HbA1c: hemoglobin A1c; TC: total cholesterol; TG: triglycerides; HDL: high-density lipoprotein; LDL: low-density lipoprotein; CVD: cardiovascular disease; DUSP1: dual-specificity phosphatase 1; hsCRP: high-sensitivity C-reactive protein; oxLDL: oxidized low-density lipoprotein.

**Table 2:** Correlation between nontraditional CVD risk markers with various parameters of the study subjects.

	DUSP1		hsCRP		oxLDL	
Parameter	Cases	Controls	Cases	Controls	Cases	Controls
Age (years)	-**0.235**^∗∗^	-0.120	0.060	-0.230	0.145	0.035
Waist (cm)	**-0.190** ^∗∗^	**0.260** ^∗^	**0.293** ^∗∗∗^	**0.250** ^∗∗^	-0.082	-0.173
Hip (cm)	-0.138	0.108	**0.383** ^∗∗∗^	**0.360** ^∗∗^	0.017	-0.034
BMI (kg/m^2^)	-0.099	**0.256** ^∗^	**0.385** ^∗∗∗^	**0.395** ^∗∗^	0.015	-0.099
SBP (mmHg)	-0.070	**0.257** ^∗^	0.137	0.127	0.018	**-0.386** ^∗^
DBP (mmHg)	0.070	0.085	0.139	0.247	**-0.167** ^∗^	-**0.392**^∗∗^
FBG (mmol/l)	-0.129	-0.001	0.011	-0.026	-0.075	0.135
TC (mmol/l)	0.117	0.108	**0.236** ^∗∗^	0.018	-0.117	**0.271** ^∗^
TG (mmol/l)	0.096	0.174	0.112	**0.272** ^∗^	**-0.395** ^∗∗∗^	**-0.365** ^∗∗∗^
HDL (mmol/l)	**-0.176** ^∗^	-0.085	0.050	-0.192	**0.354** ^∗∗∗^	**0.515** ^∗∗∗^
LDL (mmol/l)	0.111	0.030	**0.181** ^∗^	-0.017	-0.056	**0.253** ^∗^
oxLDL (*μ*g/l)	**-0.184** ^∗∗^	-0.164	0.068	-0.127 (0.332)	—	—
hsCRP (*μ*g/l)	-0.018	0.176	—	—	0.068	-0.127
DUSP1 (ng/l)	—	—	-0.018	0.176	**-0.184** ^∗∗^	-0.164

Values are Spearman's correlation coefficients. ^∗^*p* < 0.05; ^∗∗^*p* < 0.01; ^∗∗∗^*p* < 0.001.

**Table 3:** Multivariate regression models for independent associated CVD risk factors.

Effect	OR	95% CI	*p* value
Age	1.054	1.024-1.085	0.0003
LDL	0.546	0.387-0.771	0.0006
oxLDL	0.461	0.255-0.834	0.0104
hsCRP	1.511	1.059-2.154	0.0227
DUSP1	2.277	1.070-4.845	0.0328

Model: age, gender, BMI, waist, hip, SBP, DBP, TC, TG, HDL, LDL, DUSP1, hsCRP, and oxLDL are included in model.

**Table 4:** Trend analysis of the population study segregated according to statin treatment.

Parameter	Cases (statin) *n* = 111	Cases (no statin) *n* = 96	Controls *n* = 70	*p* value
Gender				
Age (years)	58.77 ± 11.7	49.63 ± 12.06	47.11 ± 11.5	**<0.0001**
Weight (kg)	86.20 ± 16.47	85.41 ± 18.40	85.60 ± 18.1	0.9452
Waist (cm)	104.88 ± 13.35	101.44 ± 14.23	99.60 ± 12.8	**0.0303**
Hip (cm)	109.14 ± 11.84	109.51 ± 12.35	110.5 ± 11.0	0.7466
BMI (kg/m^2^)	31.44 ± 5.98	31.30 ± 5.98	30.91 ± 5.36	0.8315
SBP (mmHg)	136.9 ± 17.96	136.79 ± 22.51	129.1 ± 17.7	**0.0180**
DBP (mmHg)	76.10 ± 10.57	79.71 ± 13.28	79.30 ± 11.8	**0.0563**
FBG (mmol/l)	7.87 ± 3.12	6.74 ± 3.78	4.91 ± 0.38	**<0.0001**
HbA1c	7.70 ± 1.76	6.84 ± 2.40	5.60 ± 0.46	**<0.0001**
TC (mmol/l)	4.58 ± 1.22	5.21 ± 1.29	5.42 ± 0.97	**<0.0001**
TG (mmol/l)	1.86 ± 1.53	1.64 ± 1.29	1.46 ± 0.85	0.1177
HDL (mmol/l)	1.07 ± 0.31	1.13 ± 0.40	1.21 ± 0.37	**0.0282**
LDL (mmol/l)	2.65 ± 1.05	3.33 ± 0.95	3.54 ± 0.80	**<0.0001**
DUSP1 (ng/ml)^#^	27.03 (11.25-139.08)	30.25 (11.25-100.04)	25.14 (0.33-68.65)	**0.0221** ^∗^
hsCRP (mg/l)^#^	3.00 (0.10-11.85)	3.40 (0.32-10.50)	2.19 (0.20-10.50)	**0.0537** ^∗^
oxLDL (*μ*g/l)^#^	15.37 (0.03-57.67)	19.00 (3.71-61.21)	22.77 (5.19-60.25)	**0.0017** ^∗^

^∗^Kruskal-Wallis test. ^#^Median (min-max).

**Table 5:** Trend analysis of the population study segregated according to diabetes status.

Parameter	CVD diabetic *n* = 140	CVD nondiabetic *n* = 67	Controls *n* = 70	*p* value
Gender				
Age (years)	58.24 ± 10.38	46.79 ± 10.51	47.11 ± 11.50	**<0.0001**
Weight (kg)	87.39 ± 17.37	82.60 ± 16.20	85.60 ± 18.10	0.1832
Waist (cm)	105.75 ± 14.38	98.23 ± 11.14	99.60 ± 12.84	**0.0001**
Hip (cm)	110.34 ± 12.69	107.25 ± 10.43	110.51 ± 11.04	0.1639
BMI (kg/m^2^)	31.87 ± 6.38	30.35 ± 4.89	30.91 ± 5.36	0.1798
SBP (mmHg)	139.80 ± 19.22	130.69 ± 20.78	129.1 ± 17.71	**0.0001**
DBP (mmHg)	77.29 ± 12.08	78.70 ± 11.90	79.30 ± 11.80	0.4652
FBG (mmol/l)	8.52 ± 3.68	4.90 ± 0.37	4.91 ± 0.38	**<0.0001**
HbA1c	8.10 ± 2.17	5.70 ± 0.46	5.60 ± 0.46	**<0.0001**
TC (mmol/l)	4.76 ± 1.35	5.10 ± 1.13	5.42 ± 0.97	**0.0008**
TG (mmol/l)	1.89 ± 1.41	1.48 ± 0.78	1.46 ± 0.85	**0.0242**
HDL (mmol/l)	1.06 ± 0.30	1.18 ± 0.44	1.21 ± 0.37	**0.0048**
LDL (mmol/l)	2.82 ± 1.06	3.26 ± 1.00	3.54 ± 0.80	**<0.0001**
DUSP1 (ng/ml)^#^	27.37 (11.25-139.08)	30.27 (11.25-77.61)	25.14 (0.33-68.65)	**0.0131** ^∗^
hsCRP (mg/l)^#^	3.24 (0.10-11.86)	3.10 (0.44-10.05)	2.19 (0.20-10.50)	0.0865^∗^
oxLDL (*μ*g/l)^#^	15.45 (0.03-61.21)	18.01 (2.58-54.42)	22.77 (5.19-60.25)	**0.0025** ^∗^

^∗^Kruskal-Wallis test. ^#^Median (min-max).

## Data Availability

No data were used to support this study.

## Abbreviations

DUSP1: Dual-specificity phosphatase 1
hsCRP: High-sensitivity C-reactive protein
oxLDL: Oxidized low-density lipoprotein
BMI: Body mass index
BP: Blood pressure
DBP: Diastolic blood pressure
SBP: Systolic blood pressure
FBG: Fasting blood glucose
HbA1c: Hemoglobin A1c
TG: Triglycerides
TC: Total cholesterol
LDL: Low-density lipoprotein
HDL: High-density lipoprotein
CVD: Cardiovascular disease
MAPK: Mitogen-activated protein kinase
ACE: Angiotensin-converting enzyme
REDCap: Research electronic data capture.
